# Uptake of USPSTF recommendation to refer pregnant individuals for therapy or counseling to prevent perinatal depression

**DOI:** 10.1007/s00737-022-01267-z

**Published:** 2022-09-24

**Authors:** Jennifer N. Felder, Riya Mirchandaney, Sona Dimidjian

**Affiliations:** 1 Department of Psychiatry and Behavioral Sciences, University of California, San Francisco, USA; 2 Osher Center for Integrative Health, University of California, San Francisco, UCSF, Box 1726, San Francisco, CA 94143 USA; 3 Crown Institute and Department of Psychiatry and Neuroscience, University of Colorado Boulder, Boulder, USA

**Keywords:** Perinatal depression, Prevention, Screening, Referral

## Abstract

**Purpose:**

Perinatal depression is the most common complication of pregnancy and childbirth, and it is associated with adverse consequences. The United States Preventive Services Task Force (USPSTF) recommends that pregnant and postpartum (i.e., perinatal) individuals at risk for depression be referred for therapy or counseling interventions; however, it is unclear to what extent this recommendation has been implemented.

**Methods:**

Pregnant individuals were recruited via advertisements on a pregnancy app and a separate study on sleep. Respondents completed the initial screening questions to determine their risk for perinatal depression, defined as self-reported history of depression; recent stressors; history of emotional, sexual, or physical abuse; mild depressive symptoms; anxiety symptoms; single; diabetes diagnosis; or unwanted pregnancy. Eligible respondents reported their providers’ recommendations for preventing depression, and their utilization of interventions to prevent depression (*n* = 303).

**Results:**

Fewer than 15% of participants reported that a provider referred them for therapy or counseling to prevent depression; recommendations included cognitive behavioral therapy (4%), interpersonal psychotherapy (2.3%), mindfulness-based cognitive therapy (4.3%), or other/unknown (6.6%). Approximately 12% reported that a provider recommended medication to prevent depression. Provider referral rates varied by risk factor, but not by patient demographics. Nearly 20% of participants reported using therapy or counseling to prevent depression, and nearly 13% reported using medication to prevent depression.

**Conclusions:**

We explore potential factors affecting the uptake of the USPSTF recommendation and underscore the importance of preventing perinatal depression.

**Supplementary Information:**

The online version contains supplementary material available at 10.1007/s00737-022-01267-z.

## Introduction

Perinatal depression is associated with distress, impairment, and long-term consequences such as increased risk of suicide and impairments in parenting (Khalifeh et al. 2016; Stein et al. 2014). It costs society billions of dollars due to productivity loss, increased use of welfare and Medicaid, and higher health care expenditures (Luca et al. 2019). Thus, preventive interventions may avert these negative maternal, child, and societal consequences. Fortunately, there is now strong evidence that interventions such as cognitive behavioral therapy and interpersonal psychotherapy can prevent depression during pregnancy and the postpartum period (i.e., perinatal depression) (O'Connor et al. 2019). Accordingly, in 2019, the United States Preventive Services Task Force (USPSTF) recommended that perinatal individuals at risk for depression be referred for therapy or counseling interventions (U. S. Preventive Services Task Force et al. 2019).

There is a rich body of research demonstrating that screening and referral for *current* depression in the USA are inadequate, despite recommendations by the American College of Obstetricians and Gynecologists (ACOG) and the American Academy of Pediatrics (AAP) that perinatal people be screened for current depression and referred for care. For example, in one 2020 study of perinatal women in a large US health system, 34.9% of the sample were not screened for depression at any prenatal visit (Sidebottom et al. 2021). In this sample, women insured by Medicare/Medicaid were less likely to be screened than women who were privately insured, and women of color were less likely to be screened than white women.

Given time and resource constraints, it is likely that screening for depression risk is a lower priority for providers than screening for acute depression. However, it is unknown whether the USPSTF recommendation has been translated into routine care. To address this gap, we surveyed approximately 300 pregnant people at risk for depression to assess the extent to which their providers recommended an intervention to prevent depression, whether provider recommendations varied by participant characteristics, and participant utilization of interventions to prevent depression.

## Methods

Participants were recruited via advertisements on a pregnancy app and for a separate study about sleep. Eligibility criteria were: pregnant; 18 years or older; not currently depressed (Patient Health Questionnaire-9 total ≤ 10) (Kroenke et al. 2001); and at risk for depression. Respondents were eligible if they endorsed any clinical risk factors associated with the development of perinatal depression (O'Connor et al. 2019). Per the USPSTF evidence report, these include psychological factors (e.g., history of depression or current subthreshold depressive symptoms), physical health factors (e.g., gestational diabetes), socioeconomic factors (e.g., low income or single parenthood), or a recent history of significant negative life events. A list of all risk factors and how they were ascertained is available in the Supplement. The study received approval from the institutional review board of a large public university on 29 July 2020, and all participants provided electronic informed consent.

Participant-reported characteristics, provider recommendations, and utilization were examined descriptively. Binary logistic regression and chi-squared tests were used to explore the extent to which provider recommendations varied by participant characteristics and risk type.

## Results

Of 525 individuals who completed the screening survey between April 2021 and January 2022, 303 (57.7%) were at risk for depression and eligible to participate. Participant characteristics are reported in Table 1. In brief, participants were in their early 30 s on average (M = 32.43, SD = 4.80), approximately 63% were white, and most had multiple risk factors for depression (76.2%).

**Table 1:** Participant characteristics and whether recommendations for therapy or counseling varied by characteristic

	% (*n*) or M (SD)	p value
Age (years)	32.43 (4.80)	0.80
Race/ethnicity		0.36
Asian or Pacific Islander	9.2% (28)	
Latinx, Latin American, or Hispanic	11.9% (36)	
White or European American	62.7% (190)	
African American or Black	8.9% (27)	
Native American, American Indian, Alaskan Native, or indigenous person or other	0.99% (3)	
Bi- or multi-racial/ethnic	6.3% (19)	
College graduate	69.6% (211)	0.64
Primiparous	44.9% (136)	0.12
Gestational age	24.93 (7.87)	0.06
Risk factor type		
Multiple risk factors	76.2% (231)	0.004^**^
History of depression	37.3% (113)	< 0.001^***^
Recent stressors	49.5% (150)	0.23
Emotional abuse	33.0% (100)	0.08
Sexual or physical abuse	37.0% (112)	0.65
Mild depressive symptom severity	49.5% (150)	0.03^*^
Moderate-to-severe anxiety symptom severity	12.5% (38)	0.009^**^
Single	3.0% (9)	0.75
Low income	19.1% (58)	0.14
Diabetes	11.2% (34)	0.12
Pregnancy mistimed, unwanted, or unsure	29.4% (89)	0.53

Boldface indicates statistical significance (^*^p < 0.05, ^**^p < 0.01, ^***^p < 0.001)

As reported in Table 2, approximately 15% (*n* = 45) of participants reported that a provider recommended therapy or counseling to prevent depression. Only 4% (*n* = 12) reported that a provider recommended cognitive behavioral therapy, and 2.3% (*n* = 7) reported that a provider recommended interpersonal psychotherapy. Nearly 20% reported utilizing therapy or counseling to prevent depression (*n* = 60), approximately half of which had not received this recommendation from a provider (51.7%, *n* = 31).

**Table 2:** Rates of depression risk identification, recommendations for depression prevention, and treatment utilization

Provider recommendations	
Provider talked about risk for perinatal depression	41.6% (126/303)
Provider told participant they were at higher risk for perinatal depression	14.9% (45/302)
Provider recommended therapy or counseling to prevent depression	14.9% (45/303)
Cognitive-behavior therapy	4% (12/303)
Interpersonal psychotherapy	2.3% (7/303)
Mindfulness-based cognitive therapy	4.3% (13/303)
Other/unknown^a^	6.6% (20/303)
Provider recommended medication to prevent depression	12.2% (37/303)
Antidepressant	8.6% (26/303)
Anti-anxiety	5.9 (18/303)
Other^b^	1.7% (5/303)
Provider made other recommendations for preventing depression	8.3% (25/303)
Patient utilization	
Patient used therapy or counseling to prevent depression	19.9% (60/301)
Cognitive-behavior therapy	7.9% (24/303)
Interpersonal psychotherapy	5.3% (16/303)
Mindfulness-based cognitive therapy	6.6% (20/303)
Other/unknown^c^	5.6% (17/303)
Patient used medication to prevent depression	12.7% (38/300)
Antidepressant	8.9% (27/303)
Anti-anxiety	5.3% (16/303)
Other^d^	1.3% (4/303)
Patient did anything else to prevent depression^e^	29% (88/303)

^a^e.g., family therapy, general counseling, group therapy, and monthly check in; ^b^e.g., antipsychotic and mood stabilizer; ^c^e.g., brain spotting, EMDR, family or couples counseling, memory regression, monthly check in, and psychodynamic; ^d^e.g., antipsychotic and mood stabilizer; ^e^e.g., exercise, meditation, prayer, journaling, supplements, and healthy eating

Recommendations to start or stay in therapy or counseling varied significantly by risk type (Table 1), such that those with multiple risk factors, a history of depression, mild depressive symptom severity, or moderate-to-severe anxiety symptom severity were more likely to receive a recommendation for therapy or counseling relative to those without these respective risk factors. Recommendations to start or stay in therapy or counseling did not vary by any other participant characteristic.

## Discussion

Our findings suggest that the USPSTF’s 2019 recommendation that pregnant individuals at risk for depression be referred for therapy or counseling to prevent depression has not yet been translated into clinical practice. In this sample of pregnant individuals at risk for becoming depressed, fewer than 1 in 6 reported that their provider recommended therapy or counseling to prevent depression.

Several factors may affect uptake. First, there is no single validated screening tool for identifying each of the risk factors outlined in the evidence report (O'Connor et al. 2019). Second, providers may have insufficient time to screen during brief appointments that have competing priorities. Third, providers may be reluctant to refer patients when there are significant mental health workforce shortages and limited financial coverage of preventive interventions. In order to understand how best to increase rates of screening and referral, further research is needed to understand provider-level barriers and facilitators.

These findings were underscored by participants’ contributions to the free-response section of the survey, with one participant sharing: “It doesn’t seem like they’re very educated about or necessarily concerned about the topic. I haven’t been provided any ideas to try by myself at home by any of my providers – I’ve only been told that maybe a counselor or medication would help, and that’s mostly to reduce depression once it’s started, not to help prevent it. I am only familiar with home ideas (taking certain supplements, meditation, yoga, setting relationship boundaries, getting hugs, etc.) because of my own extensive reading and research.”

## Limitations

It is possible that our recruitment methods left our dataset vulnerable to selection bias. Because we primarily recruited via advertisements on a pregnancy app, we were not able to assess the response rate of our survey. Although our recruitment advertisements did not state the study purpose, the ad text referenced “mental healthcare” and “healthcare providers,” which may have biased our sample. Additionally, 12% of our final sample was recruited from a separate study on sleep during pregnancy; people interested in a sleep study may differ from the general population of perinatal individuals. Given these recruitment methods and our sample size, there may be limits to generalizability.

There are various limitations in our assessment of provider recommendations. We relied solely on patient self-report and did not include provider-report or behavioral observation. We did not collect information about provider type or demographics; thus, it is unknown whether screening and referral rates vary among different types of providers (obstetricians, nurses, midwives, etc.).

Finally, our sample was not representative of the overall US birthing population, thus limiting the generalizability of our results. Our sample had a higher rate of non-Hispanic white respondents than the US birthing population (62.7% vs 52.1%) (US Census Bureau and National Center for Health Statistics). Another limitation to the generalizability of our results is the lack of data regarding participant location within the USA. Minors in this study did not meet the criteria of consenting for themselves; consequently, we were unable to assess adolescent parenthood as a risk factor for perinatal depression.

## Summary

Perinatal depression is the most common complication of pregnancy and childbirth and is associated with adverse consequences for both mother and infant (Grote et al. 2010; Stein et al. 2014). Regular screenings and referrals for preventing perinatal depression, as recommended by USPSTF, may be a clinically and cost-effective method of mitigating these consequences, but our findings suggest that there may be low uptake of this recommendation. Further research is needed to identify and target patient-, provider-, and system-level barriers to implementing USPSTF recommendations to prevent perinatal depression.

## Supplementary Information

Below is the link to the electronic supplementary material.

Supplementary file1 (DOCX 19 KB)

## Data Availability

All data are available from the PI upon reasonable request for data access.
