# Supplementary material for: Uptake of USPSTF recommendation to refer pregnant individuals for therapy or counseling to prevent perinatal depression
Source: Arch Womens Ment Health. 2022 Sep 24;25(6):1149–53. doi: 10.1007/s00737-022-01267-z (PMC9734199; doi:10.1007/s00737-022-01267-z)
Supplement: Supplementary file 1 — Supplementary file1 (DOCX 19 KB) [file 737_2022_1267_MOESM1_ESM.docx]

**Online Supplementary Table**. Items used to ascertain risk for perinatal depression

| Construct | Measure/Item | Included if |
| --- | --- | --- |
| History of depression | Have you ever had a period of 2 weeks or more when you felt particularly worried, miserable, or depressed? (Item derived the Antenatal Risk Questionnaire)  **No/Yes** | “Yes” *and* |
|  | If **yes**, did this: |  |
|  | Seriously interfere with your work or your relationships with friends and family?  **Not at all; A little; Somewhat; Quite a lot; Very much** | “Quite a lot” or “Very much” *or* |
|  | Lead you to seek professional help?  **No/Yes** | “Yes” |
| Recent stressors | Have you had any stresses, changes, or losses in the last 12 months? (examples: separation, domestic violence, job loss, bereavement, etc.; Item derived the Antenatal Risk Questionnaire)  **No/Yes** | “Yes” |
| Emotional abuse | Were you emotional abused *when you were growing up?* (Item derived the Antenatal Risk Questionnaire)  **No/Yes** | “Yes” |
| Sexual or physical abuse | Have you ever been sexually or physically abused? (Item derived the Antenatal Risk Questionnaire)  **No/Yes** | “Yes” |
| Mild depressive symptom severity | Patient Health Questionnaire-9 (PHQ-9) | Total score = 5-9 |
| Moderate-to-severe anxiety symptom severity | Generalized Anxiety Disorder Scale-7 (GAD-7) | Total score ≥ 10 |
| Single | Which of the following best describes your relationship status?  **Married or living with partner; Significantly involved with a partner, but not living together; Single/not significantly involved with a partner** | “Single/not significantly involved with a partner” |
| Low income | What is your yearly household income before taxes? How many people, including you, live on that income? | ≤ 150% of 2019 federal poverty line |
| Diabetes | *During* your current pregnancy, were you diagnosed with gestational diabetes (diabetes that started *during* this current pregnancy)?  *Before* your current pregnancy, were you diagnosed with pregestational diabetes (diabetes that started *before* this current pregnancy)?  **No/Yes** | “Yes” to either |
| Pregnancy mistimed, unwanted, or unsure | Thinking back to just before you got pregnant this time, how did you feel about becoming pregnant? Check ONE answer. (Item derived the Pregnancy Risk Assessment Monitoring System)  **I wanted to be pregnant later; I wanted to be pregnant sooner; I wanted to be pregnant then; I didn’t want to be pregnant then or at any time in the future; I wasn’t sure what I wanted** | “I wanted to be pregnant later” *or* “I didn’t want to be pregnant then or at any time in the future” *or* “I wasn’t sure what I wanted” |

*Note.* Respondents only needed to have one risk factor to qualify for participation in this survey study.
